# Novel Treatment Paradigms: Focal Segmental Glomerulosclerosis

**DOI:** 10.1016/j.ekir.2022.10.004

**Published:** 2022-10-08

**Authors:** Marina de Cos, Kristin Meliambro, Kirk N. Campbell

**Affiliations:** 1 Division of Nephrology, Department of Medicine, Icahn School of Medicine at Mount Sinai, New York, New York, USA

**Keywords:** FSGS, Glomerular Disease, Treatment

## Abstract

Focal segmental glomerulosclerosis (FSGS) is a histologic pattern of injury defined by the presence of sclerosis in some (segmental) of certain glomeruli (focal). On electron microscopy, it is characterized by a variable degree of podocyte foot process effacement and gaps in the coverage of the glomerular basement membrane. The pattern of injury occurs when podocytes, highly differentiated cells with limited regenerative capacity, are reduced in number. The heterogeneity in underlying causes of podocyte loss results in equally variable clinical phenotypes. Recent work acknowledging advances in defining the genetic and immunologic basis of disease has redefined the classification of FSGS. Unprecedented clinical trial activity and efficacy of repurposed agents presents hope for improved therapeutic options. This minireview summarizes recent advances with a focus on novel treatment paradigms in FSGS.

### Current Treatment Guidelines

As a pattern of injury and not a disease, the histologic finding of FSGS in a kidney biopsy is considered the beginning of a process to identify a specific and hopefully treatable underlying cause. Appropriate treatment decisions are based on sound clinical entity characterization. Contemporary classification relies on integrating the clinical history, laboratory results, kidney biopsy, and genetic testing results, among other data sources. FSGS cases can be divided into 4 categories: (i) those resulting from an immunologic cause, thought to be a circulating glomerular permeability factor (defined as primary); (ii) those that occur secondary to a systemic process known to cause FSGS (including maladaptive FSGS, viral or drug-induced FSGS); (iii) those caused by a genetic mutation in a podocyte or glomerular basement membrane protein; and (iv) those that occur in the absence of an identifiable cause but seem to be unrelated to a circulating permeability factor (or FSGS of undetermined cause).^1^ Stratification of patients into these groups is sometimes challenging because different causes and risk factors can overlap to reach the threshold of podocyte injury that leads to FSGS. In addition, patients designated as having FSGS–undetermined cause may have as yet unidentified secondary or genetic underpinnings. No light microscopic changes distinguish the subtypes, but some unique clinical and pathologic features have been identified. Patients with primary FSGS typically have abrupt-onset marked proteinuria and overt nephrotic syndrome with diffuse podocyte foot process effacement on electron microscopy.^2^ Glomerulomegaly is very common in FSGS secondary to obesity, reflux nephropathy, and individuals with low birth weight, but it can also be seen in primary disease.^3^ Genetic FSGS can have variable clinicopathologic features but is more common in patients with a family history of glomerular disease and in those resistant to glucocorticoids.

The recently updated Kidney Disease: Improving Global Outcomes practice guidelines for the treatment of FSGS recommend supportive treatment for all patients with persistent proteinuria with the use of renin angiotensin aldosterone system (RAAS) blockade, optimal blood pressure control, and dietary salt restriction.^1^ Although RAAS blockade reduces proteinuria in patients with FSGS,^4^ additional agents are typically required to achieve a sustained clinical remission. The use of sodium glucose cotransporter 2 inhibitor therapy has emerged as an attractive supportive agent class used in conjunction with RAAS inhibitors for across the spectrum of proteinuric kidney disease. Prespecific analyses of the Dapagliflozin and Prevention of Adverse Outcomes in Chronic Kidney Disease study have shown protective benefits of dapagliflozin from estimated glomerular filtration rate decline in IgA nephropathy and FSGS, although the effect was not statistically significant in the FSGS substudy.^5^^,^^6^ Future studies will be needed to further define the benefit of sodium glucose cotransporter 2 inhibitor in FSGS. An additional drug class likely to be considered for supportive therapy is nonsteroidal mineralocorticoid receptor antagonists. These agents have anti-inflammatory and antifibrotic properties with finerenone, a nonsteroidal mineralocorticoid receptor antagonist now approved by the US Food and Drug Administration for diabetic kidney disease.^7^

Kidney Disease: Improving Global Outcomes guidelines recommend that patients with clinical and histologic features of primary FSGS, including the presence of nephrotic syndrome, be treated with high-dose immunosuppression (initially with high-dose glucocorticoids) as first-line therapy. Patients who fail to respond to glucocorticoids or those with a contraindication to their use are treated with calcineurin inhibitors.^1^ Alternative therapies such as mycophenolate mofetil, adrenocorticotropic hormone, or rituximab, have been used over time with different degrees of success, but their efficacy is still debatable and have therefore not been included in the current guidelines. Extracorporeal therapies such as plasma exchange, immunoadsorption, and low-density lipoprotein apheresis may have a role as adjunctive therapy for patients who fail to respond to steroids and other immunosuppressive agents.^8^ In the absence of robust randomized clinical trial data, patient selection criteria, the optimal apheresis approach, and concomitant immunosuppressive regimens remain unclear. For those patients with secondary FSGS, treatment is focused on the underlying condition.^1^ A summary of Kidney Disease: Improving Global Outcomes guidelines treatment recommendations based on FSGS etiology can be found in Figure 1.

**Figure 1:**
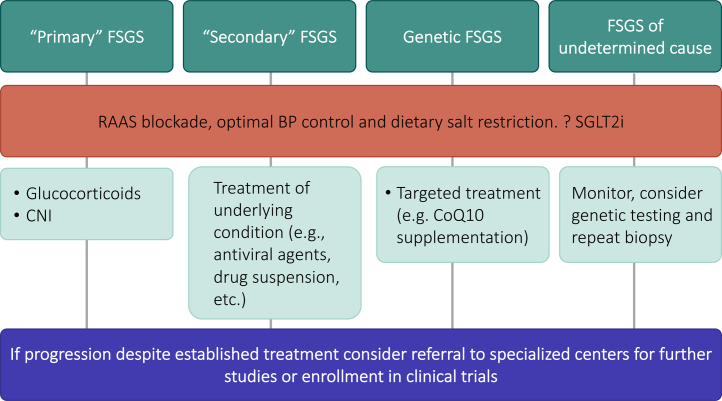
Current treatment of FSGS based on KDIGO practice guideline recommendations. BP, blood pressure; CNI, calcineurin inhibitor; CoQ10, coenzyme Q_10_; FSGS, focal segmental glomerulosclerosis; KDIGO, Kidney Disease: Improving Global Outcomes; RAAS, renin angiotensin aldosterone system; SGLT2i, sodium glucose cotransporter 2 inhibitor.

### Emerging Treatment Options

Despite translational research advances and increased knowledge regarding the pathogenesis of FSGS, targeted therapies are still lacking, and treatment strategies have not changed significantly over the past several decades. Nevertheless, there has recently been a notable increase in emerging therapies targeting defined pathogenic signaling cascades. A summary of recent and ongoing clinical trials for FSGS is provided in Table 1. A brief, and not comprehensive, discussion of some of these promising emerging agents (Figure 2) and representative signaling pathways they target will now be discussed.

**Table 1:** Some recent and ongoing clinical trials in focal segmental glomerulosclerosis

NCT number	Drug	Mechanism of action	Status	Phase	Completion
NCT01613118	Sparsentan	Dual ETA receptor/AT1 receptor antagonist	Active, not recruiting	Phase 2	February 2026
NCT03493685				Phase 3	
NCT05003986			Peds: Recruiting	Phase 2	June 2025
NCT04573920	Atrasentan	Dual ETA receptor/AT1 receptor antagonist	Recruiting	Phase 2	February 2026
NCT03970122	GFB-887	*TRPC5* channel inhibitor	Completed	Phase 1	April 2020
NCT04387448			Recruiting	Phase 2	August 2022
NCT04950114				Phase 2	September 2025
NCT03448692	PF-067301512	*SLIT2* antagonist	Recruiting	Phase 2	August 2025
NCT04340362	VX-147	*APOL1* antagonist	Completed	Phase 2	December 2021
NCT05312879			Recruiting	Phase 2/3	June 2026
NCT05267262	R3R01	Lipid-modifying drug	Not yet recruiting	Phase 2	December 2023
NCT05213624	BI764198	*TRPC6* inhibitor	Recruiting	Phase 2	August 2023
NCT05183646	DMX-200 (repagermanium)	*CCR2* inhibitor	Recruiting	Phase 3	June 2026
NCT05314231	ALXN1720	Anti-C5 mini-body	Not yet recruiting	Phase 1	March 2023
NCT05237388	Baricitinib	Janus kinase-*STAT* inhibitor	Not yet recruiting	Phase 2	March 2026
NCT00814255	Adalimumab	Antihuman TNF-α antibody	Completed	Phase 2	February 2014
NCT04009668			+TR-MCD: Recruiting	Phase 2	July 2024
NCT05441826	VB119	Anti-CD19 antibody	Recruiting	Phase 2	February 2024
NCT04983888	Obinutuzumab	Anti-CD20 antibody	Recruiting	Phase 2	September 2024

AT1, angiotensin II receptor type 1 ; CD, cluster of differentiation; ETA, endothelin type A; MCD, minimal change disease; TNF-α, tumor necrosis factor-α.

**Figure 2:**
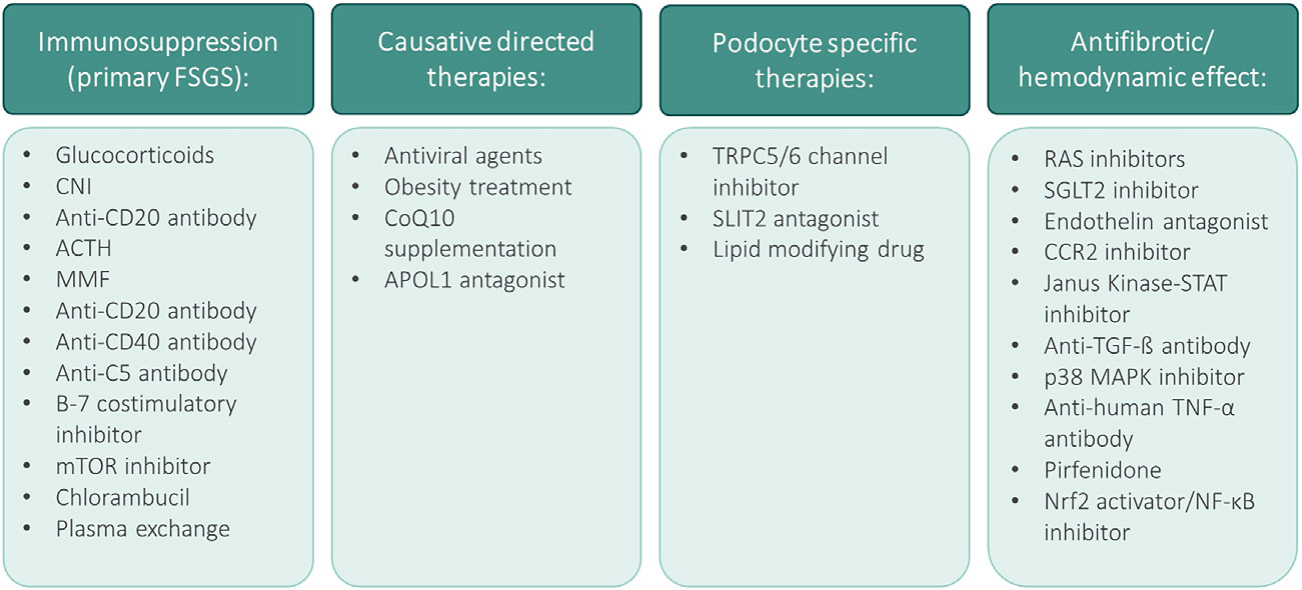
Treatment of FSGS organized by class, including both established and emerging therapeutic options. ACTH, adrenocorticotropic hormone; CCR2, C-C chemokine receptor 2; CNI, calcineurin inhibitor; FSGS, focal segmental glomerulosclerosis; MAPK, mitogen-activated protein kinase; mTOR, mammalian target of rapamycin; NF-κB, nuclear factor κB; RAS, renin-angiotensin system; SGLT2i, sodium glucose cotransporter 2 inhibitor; STAT, signal transducer and activator of transcription; TNF, tumor necrosis factor; TGF, transforming growth factor.

#### Targeting the Podocyte

Podocytes rely on a complex cytoskeletal repertoire consisting of cortical actin and a central actin bundle anchored to the glomerular basement membrane to maintain normal morphology. Disrupted intracellular calcium homeostasis is a key event in podocyte injury. Increased intracellular podocyte calcium results in activation of the calcium-dependent phosphatase calcineurin leading to dephosphorylation of the actin-bundling protein synaptopodin, rendering it susceptible to cathepsin L–mediated cleavage. Gain-of-function disease-causing mutations in *TRPC6*, associated with enhanced intracellular calcium levels, were identified in 2005 in familial FSGS.^9^ *TRPC* family members activate small GTPases in podocytes with *TRPC6* and *TRPC5* activating *RhoA* and *Rac1*, respectively. Rac1 activation further induces *TRPC5* translocation to the podocyte membrane, resulting in a Rac1-TRPC5 feed-forward loop. *TRPC6* inhibition is a potential therapeutic strategy for FSGS (NCT05213624), whereas GFB-887, an inhibitor of *TRPC5*, is in clinical trials for FSGS, minimal change disease, and diabetic nephropathy.^10^

Endothelin-1 has numerous pathogenic effects on the glomerulus, including vasoconstriction and mediation of intraglomerular hypertension and promotion of inflammation and fibrosis.^11^ In addition, angiotensin II and endothelin-1 can synergistically promote podocyte apoptosis and reorganization of the actin cytoskeleton. Earlier studies investigating dual blockade of endothelin-1 and RAAS in mouse models of membranous nephropathy and diabetic nephropathy demonstrated reduction of proteinuria, glomerular sclerosis, and tubulointerstitial damage compared with monotherapies.^11^ Sparsentan, a dual endothelin type A and angiotensin II type 1 receptor antagonist, was shown in a phase 2, randomized, double-blind, active-control study of patients with FSGS to decrease proteinuria from baseline by 45% versus 19% for patients on irbesartan control over 8 weeks (*P* = 0.006).^12^ Sparsentan is currently being further evaluated in that study's open-label treatment period and in a randomized, multicenter, double-blind, parallel, active-control phase 3 study (NCT03493685).^13^ Atrasentan is an endothelin type A receptor antagonist without angiotensin II type 1 receptor inhibitory properties that is being studied in a phase 2, open-label basket study for patients with FSGS in addition to IgA nephropathy, Alport syndrome, and diabetic nephropathy (NCT04573920). Atrasentan reduced the risk of renal events in patients with diabetes and chronic kidney disease in the phase 3 randomized, placebo-controlled SONAR trial when used in conjunction with maximum labeled or tolerated renin-angiotensin system inhibition.^14^ Thus, endothelin-1/endothelin type A inhibition may provide opportunities to potentially enhance podocyte protection beyond RAAS blockade.

The *ROBO2* colocalizes with the podocyte slit diaphragm protein nephrin and is a receptor for *SLIT2*. *ROBO2*/*SLIT2* signaling has been shown to reduce podocyte focal adhesions and decrease podocyte adhesion to collagen-coated plates *in vitro*.^15^ Podocyte *ROBO2* expression is increased in mice and humans with glomerular disease, and loss of *ROBO2 in vivo* was protective in glomerular injury models as assessed by preservation of podocyte foot processes and reduction of proteinuria.^16^ The *ROBO2* fusion protein (PF-067301512) inhibits *ROBO2*/*SLIT2* signaling with the efficacy, safety, tolerability, and pharmacokinetics profile of PF-06730512 currently being investigated in a phase 2a, open-label, multicenter study in adult patients with FSGS (NCT03448692).^17^

Recent evidence suggests that accumulation of renal lipids may contribute to the development and progression of kidney disease and that podocytes are most susceptible to lipid-induced injury.^18^ Cholesterol efflux from podocytes is mediated in part by the transmembrane *ABCA1*.^19^ In addition, microarray analysis of mRNA isolated from glomeruli of patients with FSGS in the Nephrotic Syndrome Study Network study^20^ revealed that key genes regulating cholesterol homeostasis were differentially expressed in patients with FSGS compared with normal living donor controls.^21^ Abnormalities in lipid metabolism have also been demonstrated in other experimental models of glomerular disease, including diabetic nephropathy and Alport syndrome.^22^ These studies and others have led to the targeting of abnormal kidney and podocyte lipid accumulation as a novel therapeutic strategy for glomerular diseases. R3R01 is a small molecule designed to decrease renal lipid content, and a future phase 2, multicenter, open-label study is currently planned to evaluate the safety, efficacy, and pharmacokinetics profile of R3R01 in patients with steroid-resistant FSGS and patients with Alport syndrome on angiotensin-converting enzyme/angiotensin receptor blocker therapy with uncontrolled proteinuria (NCT05267262).

#### Precision-Based Approaches Based on Gene Sequencing

FSGS is included on the spectrum of *APOL1*-associated kidney disease, which encompasses chronic kidney disease occurring in patients with 2 “high-risk” mutations (either G1/G1, G1/G2, or G2/G2) in the *APOL1* gene.^23^ These *APOL1* mutations are found exclusively in patients of African ancestry, and recent genome-wide association studies have demonstrated that the presence of 2 *APOL1* mutations can account for up to 70% of nondiabetic kidney disease in these individuals.^24^ Potential mechanisms for *APOL1*-induced podocyte injury are related to the pore-forming ability of *APOL1*, which evolved as a protective mechanism against parasitic infection by (*Trypanosoma brucei brucei*) in Sub-Saharan Africa, and may contribute to abnormal ion flux leading to podocyte cell death.^25^ VX-147, an oral small-molecule inhibitor of *APOL1*, was initially tested in an open-label, single-arm, phase 2a study (NCT04340362) in patients with biopsy-proven FSGS and 2 *APOL1* high-risk alleles. Published results are pending, with a phase 2/3 adaptive, double-blind, placebo-controlled study using the same agent planned among a broader patient population with proteinuria and *APOL1*-associated kidney disease (NCT05312879). Alternative approaches have been proposed for *APOL1* inhibition including the use of antisense oligonucleotides^26^ and JAK-STAT inhibition with baricitinib (NCT05237388) for cytokine-induced *APOL1*-mediated podocytopathy.^27^

Targeted therapy for patients with *APOL1* high-risk alleles would be an important advance in precision therapeutics for FSGS and an illustration of the benefits of genetic testing for a condition where at least 20% of individuals with steroid-resistant disease have monogenetic etiologies.^28^ This list, now greater than 50, notably does not include disease modifiers such as *APOL1* that require a “second hit” for a clinical phenotype. A clear case for genetic testing for monogenic causes is the identification of rare recessive mutations in genes such as *COQ2*, *COQ6*, and *ADCK4* involved in coenzyme Q_10_ biosynthesis in patients with nephrotic syndrome in whom coenzyme Q_10_ supplementation may lead to clinical improvement.^29^ A summary of emerging therapeutics and their mechanism of action is illustrated in Figure 3.

**Figure 3:**
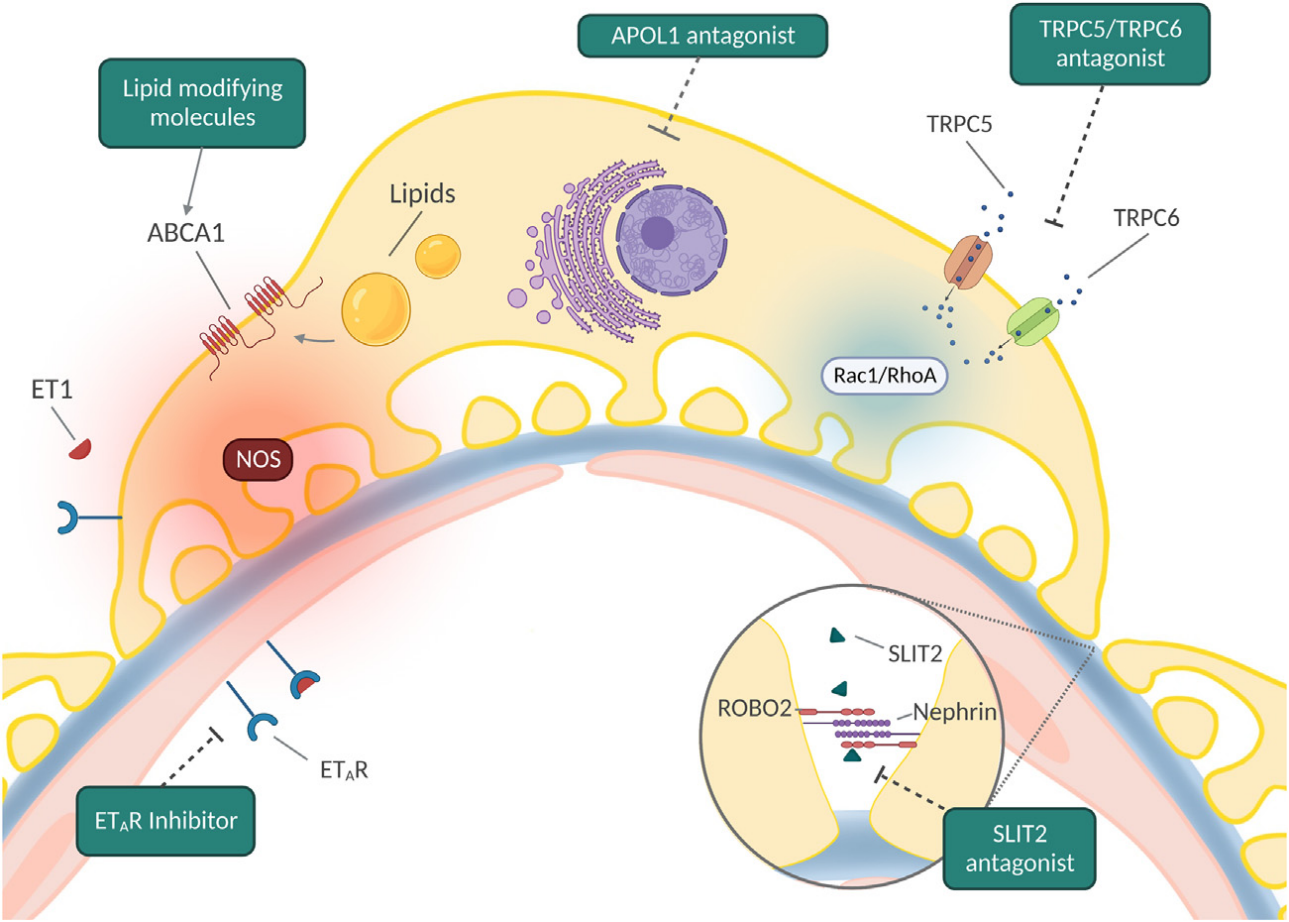
Promising emerging therapeutic targets and their mechanism of action in the treatment of focal segmental glomerulosclerosis. ET1, endothelin-1; ETAR, endothelin receptor type A; NOS, nitric oxide synthase.

#### Defining Treatment Success

There has been a lack of consensus in defining remission end points in FSGS. Traditionally, in glomerular disease clinical management and trials, a complete remission has been a reduction of proteinuria to <0.3 g/d with a stable serum creatinine and serum albumin >3.5 g/dl, whereas a partial remission is a reduction of proteinuria to 0.3 to 3.5 g/d and a decrease >50% from baseline. These definitions are however not evidence based for FSGS. Complete remissions, although ideal, are rarely achieved. Recently, an outcomes-based definition of partial remission based on data from 466 patients with primary FSGS across 5 independent cohorts has been proposed. Achieving either a complete remission or this novel definition of partial remission (40% proteinuria reduction and proteinuria <1.5 g/g) is associated with better long-term kidney survival in patients with FSGS.^30^ Further validation and adoption of this metric would be helpful to clinicians and, if approved by regulatory agencies, could facilitate more efficient randomized controlled trial end points in FSGS drug development.

#### Future Directions

As a classic clinical podocytopathy, progress in FSGS in the past 2 decades has been driven mainly by a better understanding of the molecular architecture of the glomerular filtration barrier through the identification of disease-causing and disease-associated gene variants. Improved understanding of the pathogenic pathways dysregulated by these and as-yet unidentified variants will hopefully advance the quest for targeted and precision-based therapeutics. Clinical trial activity in the FSGS space has expanded remarkably, although studies largely enroll patients who passed on the histologic definition of this heterogeneous entity, rather than biomarker or mechanistic criteria that have been successful in other fields, most notably oncology. An example of the potential path forward lies in the phase 2 study of the tumor necrosis factor-α inhibitor adalimumab (NCT04009668) in patients with increased urinary excretion of biomarkers of tumor necrosis factor activation (*MCP1*/Cr and/ or *TIMP1*/Cr) at study screening.

Another area requiring clarity in the years ahead is the value of multitarget therapy. Current guidelines and practices usually recommend a stepwise algorithm with 1 potential disease-modifying agent used at a time. For patients at high risk for progression and more broadly, to shift expectations to curative therapy and sustained complete remissions, multitargeted approaches will likely be required.

Perhaps the biggest unmet diagnostic and therapeutic needs are for primary FSGS. Although evidence supports a central role for a glomerular permeability factor or factors, their composition, mechanism of action, origin, and target cells remain undefined, severely limiting diagnostic and therapeutic capabilities. Findings from National Institutes of Health–funded consortia such as Cure Glomerulonephropathy Network,^31^ Nephrotic Syndrome Study Network,^20^ and the Kidney Precision Medicine Project^32^ may identify novel biomarkers, cellular subpopulations, and signaling pathways in the quest for targeted therapeutics.

## Disclosure

KNC reports consulting fees from Travere, Goldfinch, Mallinckrodt, Chinook, ANI, and Aurinia and reports funds to his department for being a site principal investigator for studies sponsored by Mallinckrodt, Vertex, and Travere outside the submitted work. KM reports funds to her department for being a site principal investigator for studies sponsored by Goldfinch. MC declares no competing interests.

## References

[1] Kidney Disease: Improving Global Outcomes (KDIGO) Glomerular Diseases Work Group (2021). KDIGO 2021 Clinical Practice Guideline for the Management of Glomerular Diseases. Kidney Int.

[2] De Vriese A.S., Sethi S., Nath K.A. (2018). Differentiating primary, genetic, and secondary FSGS in adults: a clinicopathologic approach. J Am Soc Nephrol.

[3] Kambham N., Markowitz G.S., Valeri A.M. (2001). Obesity-related glomerulopathy: an emerging epidemic. Kidney Int.

[4] Campbell K.N., Pennese N., Zaffalon A. (2022). Efficacy and safety of ACE inhibitor and angiotensin receptor blocker therapies in primary focal segmental glomerulosclerosis treatment: a systematic review and meta-analysis. Kidney Med.

[5] Wheeler D.C., Toto R.D., Stefansson B.V. (2021). A pre-specified analysis of the DAPA-CKD trial demonstrates the effects of dapagliflozin on major adverse kidney events in patients with IgA nephropathy. Kidney Int.

[6] Wheeler D.C., Jongs N., Stefansson B.V. (2022). Safety and efficacy of dapagliflozin in patients with focal segmental glomerulosclerosis: a prespecified analysis of the dapagliflozin and prevention of adverse outcomes in chronic kidney disease (DAPA-CKD) trial. Nephrol Dial Transplant.

[7] Agarwal R., Kolkhof P., Bakris G. (2021). Steroidal and non-steroidal mineralocorticoid receptor antagonists in cardiorenal medicine. Eur Heart J.

[8] Raina R., Wang J., Sharma A., Chakraborty R. (2020). Extracorporeal therapies in the treatment of focal segmental glomerulosclerosis. Blood Purif.

[9] Winn M.P., Conlon P.J., Lynn K.L. (2005). A mutation in the TRPC6 cation channel causes familial focal segmental glomerulosclerosis. Science.

[10] Walsh L., Reilly J.F., Cornwall C. (2021). Safety and efficacy of GFB-887, a TRPC5 channel inhibitor, in patients with focal segmental glomerulosclerosis, treatment-resistant minimal change disease, or diabetic nephropathy: TRACTION-2 trial design. Kidney Int Rep.

[11] Komers R., Plotkin H. (2016). Dual inhibition of renin-angiotensin-aldosterone system and endothelin-1 in treatment of chronic kidney disease. Am J Physiol Regul Integr Comp Physiol.

[12] Trachtman H., Nelson P., Adler S. (2018). DUET: A phase 2 study evaluating the efficacy and safety of Sparsentan in patients with FSGS. J Am Soc Nephrol.

[13] Komers R., Diva U., Inrig J.K. (2020). Study design of the phase 3 Sparsentan versus irbesartan (Duplex) study in patients with focal segmental glomerulosclerosis. Kidney Int Rep.

[14] Heerspink H.J.L., Parving H.H., Andress D.L. (2019). Atrasentan and renal events in patients with type 2 diabetes and chronic kidney disease (SONAR): a double-blind, randomised, placebo-controlled trial. Lancet.

[15] Fan X., Yang H., Kumar S. (2016). SLIT2/ROBO2 signaling pathway inhibits nonmuscle myosin IIA activity and destabilizes kidney podocyte adhesion. JCI Insight.

[16] Pisarek-Horowitz A., Fan X., Kumar S. (2020). Loss of roundabout guidance Receptor 2 (Robo2) in podocytes protects adult mice from glomerular injury by maintaining podocyte foot process structure. Am J Pathol.

[17] Beck L.H., Berasi S.P., Copley J.B. (2021). PODO: trial design: phase 2 study of PF-06730512 in focal segmental glomerulosclerosis. Kidney Int Rep.

[18] Ge M., Merscher S., Fornoni A. (2021). Use of lipid-modifying agents for the treatment of glomerular diseases. J Pers Med.

[19] Ducasa G.M., Mitrofanova A., Mallela S.K. (2019). ATP-binding cassette A1 deficiency causes cardiolipin-driven mitochondrial dysfunction in podocytes. J Clin Invest.

[20] Gadegbeku C.A., Gipson D.S., Holzman L.B. (2013). Design of the nephrotic syndrome Study Network (Neptune) to evaluate primary glomerular nephropathy by a multidisciplinary approach. Kidney Int.

[21] Mitrofanova A., Molina J., Varona Santos J. (2018). Hydroxypropyl-beta-cyclodextrin protects from kidney disease in experimental Alport syndrome and focal segmental glomerulosclerosis. Kidney Int.

[22] Liu X., Ducasa G.M., Mallela S.K. (2020). Sterol-O-acyltransferase-1 has a role in kidney disease associated with diabetes and Alport syndrome. Kidney Int.

[23] Genovese G., Friedman D.J., Ross M.D. (2010). Association of trypanolytic ApoL1 variants with kidney disease in African Americans. Science.

[24] Freedman B.I., Cohen A.H. (2016). Hypertension-attributed nephropathy: what’s in a name?. Nat Rev Nephrol.

[25] Olabisi O.A., Heneghan J.F. (2017). APOL1 nephrotoxicity: what does ion transport have to do with it?. Semin Nephrol.

[26] Aghajan M., Booten S.L., Althage M. (2019). Antisense oligonucleotide treatment ameliorates IFN-gamma-induced proteinuria in APOL1-transgenic mice. JCI Insight.

[27] Nystrom S.E., Li G., Datta S. (2022). JAK inhibitor blocks COVID-19 cytokine-induced JAK/STAT/APOL1 signaling in glomerular cells and podocytopathy in human kidney organoids. JCI Insight.

[28] Yao T., Udwan K., John R. (2019). Integration of genetic testing and pathology for the diagnosis of adults with FSGS. Clin J Am Soc Nephrol.

[29] Atmaca M., Gulhan B., Korkmaz E. (2017). Follow-up results of patients with ADCK4 mutations and the efficacy of CoQ10 treatment. Pediatr Nephrol.

[30] Troost J.P., Trachtman H., Nachman P.H. (2018). An outcomes-based definition of proteinuria remission in focal segmental glomerulosclerosis. Clin J Am Soc Nephrol.

[31] Mariani L.H., Bomback A.S., Canetta P.A. (2019). CureGN study rationale, design, and methods: establishing a large prospective observational study of glomerular disease. Am J Kidney Dis.

[32] de Boer I.H., Alpers C.E., Azeloglu E.U. (2021). Rationale and design of the Kidney Precision Medicine Project. Kidney Int.

